# Temporal influences in non-elective cesarean sections and indication patterns

**DOI:** 10.1007/s00404-026-08604-y

**Published:** 2026-11-12

**Authors:** Anita C. Windhorst, Friederike S. Tönnies, Aline Wolter, Frank Oehmke, Ivo Meinhold-Heerlein, Roland Axt-Fliedner

**Affiliations:** 1 Institute of Medical Informatics, Justus Liebig University Giessen, Giessen, Germany; 2 Center for Prenatal Medicine and Fetal Therapy, University Hospital Giessen and Marburg, Giessen Site, Justus Liebig University, Giessen, Germany; 3 Department of Gynecology and Obstetrics, University Hospital Giessen and Marburg, Giessen Site, Justus Liebig University, Giessen, Germany

**Keywords:** Decision threshold, Soft indicator, Hard indicator, Obstetric quality improvement

## Abstract

**Purpose:**

Elective (pre-labour) cesarean sections are, by design, concentrated during staffed hours, limiting the interpretability of overall temporal patterns. We examined whether the proportion of documented discretion-susceptible indications for non-elective (intrapartum) cesarean sections shifts with time of day and weekday, using the Robson classification for stratification.

**Methods:**

We retrospectively analysed 5,850 cesarean sections at University Hospital Giessen and Marburg, 2015–2021, classified as elective (*n*=2,851) or non-elective (*n*=2,998) by documented decision timepoint. Among non-elective cases, we assessed threshold-susceptible ("soft") indications (protracted labour/arrest, non-reassuring fetal heart rate) by shift and weekend, adjusted for Robson group and gestational age. Guideline-defined absolute ("hard") indications served as a supportive comparison, with Holm correction for exploratory comparisons.

**Results:**

Among non-elective cesarean sections, the proportion with documented discretion-susceptible indications was significantly higher during evening/night shifts (OR 2.63, 95%CI 2.20–3.14, *p*<0.001) and, less markedly, on weekends (OR 1.37, 95%CI 1.12–1.67, *p*=0.002), consistent across all five individual indicators for shift, and across two of five for weekend. Indications with limited clinical discretion showed no comparable variation (shift OR 0.99, *p*=0.92; weekend OR 0.77, *p*=0.16), except for placenta previa (Holm-*p*=0.023), discussed as a qualification rather than a validation of the primary interpretation.

**Conclusion:**

The proportion of non-elective cesarean sections with documented discretion-susceptible indications was higher during evening/night shifts and weekends, while guideline-defined indications with limited discretion showed comparatively little variation. Because a population-based at-risk denominator was unavailable, these findings describe differences in documented indication composition among cesarean deliveries that occurred, rather than evidence that the intervention threshold itself changed. Whether this reflects clinical decision-making, documentation practice, or unmeasured case-mix factors warrants further investigation.

**Supplementary Information:**

The online version contains supplementary material available at https://doi.org/10.1007/s00404-026-08604-y.

## What does this study adds to the clinical work

**Table:** 

*What is already known: * Cesarean section rates and the timing of elective procedures vary by weekday and shift, largely reflecting scheduling during staffed working hours; whether the threshold for intervention during labour itself varies with staffing-related temporal factors has been suggested but rarely tested directly against a specific, indication-level comparison. *What this study adds: *Among non-elective cesarean sections, the proportion of documented discretion-susceptible indications (protracted labour, non-reassuring fetal heart rate patterns) was higher during evening/night shifts and, to a lesser extent, on weekends; guideline-defined indications with limited discretion showed little corresponding variation. These findings do not establish a causal change in the intervention threshold but identify a pattern in indication documentation that may warrant institutional review of decision-making and documentation practices for discretion-susceptible indications during evening/night shifts and weekends.	

## Background

Cesarean section rates in Germany have reached 30.9%, with substantial regional variation [1]. The decision for cesarean section can be made electively (primary cesarean section, before labour onset) or during labour (secondary cesarean section) or in cases of acute compromise (emergency cesarean section) [2–4].

Because elective cesarean sections are, by design, scheduled during staffed working hours, their temporal concentration on weekdays and daytime is largely definitional and reflects scheduling logistics rather than clinical decision-making. A more clinically relevant question concerns non-elective cesarean sections, where the timing of delivery is not planned and the decision to intervene is made under variable staffing conditions. Prior work suggests that the timing of unplanned cesarean section is influenced by factors unrelated to the underlying obstetric course. Costa-Ramón et al. found that physician demand for leisure time is a significant predictor of unplanned cesarean sections, distinguishing indications with an unambiguous clinical basis from a "gray area" of diagnoses, such as dystocia, where the decision is more discretionary [5]. Using a population-based at-risk denominator, Son et al. more recently found that the frequency of intrapartum cesarean delivery, including specifically for dystocia, rises throughout the day and declines at night, indicating that the overall likelihood of intervention and the character of the indications leading to it may vary independently and require separate examination [6]. Whether such variation reflects differences in staffing levels or the availability of senior obstetric expertise remains insufficiently examined: the presence of senior obstetricians on the labour ward has been associated with only small differences in intervention rates and no significant differences in neonatal outcomes [7], suggesting that seniority alone does not fully account for temporal variation in obstetric decision-making, and whether it can be distinguished from objectively acute, non-deferrable indications within the same cohort remains untested.

The Robson Ten Group Classification System, recommended by WHO for standardized analysis and international comparison of cesarean section rates [8–10], enables stratification by obstetric characteristics that determine baseline cesarean risk, independent of temporal factors. This stratification is essential for distinguishing genuine temporal effects on decision-making from differences in case mix across time periods, and for identifying which patient groups drive any observed pattern.

We, therefore, examined, within non-elective cesarean sections, whether the frequency of threshold-susceptible (discretionary) indications, as opposed to guideline-defined absolute indications, varies with time of day or day of week, stratified by Robson group.

### Study objectives

This retrospective study investigated whether the decision character of non-elective cesarean section varies by shift and day of week by: (1) restricting analysis to non-elective (intrapartum) cesarean sections, classified by documented decision timepoint rather than organizational cesarean-section type, which agreed with the latter in 93.0% of records; (2) examining the frequency of threshold-susceptible ("soft") indications, defined as protracted labour/arrest and non-reassuring fetal heart rate patterns, as markers of decision threshold, stratified by the Robson classification; (3) using guideline-defined absolute ("hard") indications, per the S3 guideline on cesarean section (AWMF 015–084), as a supportive comparison, since their occurrence cannot plausibly vary with staffing-related temporal factors; and (4) performing multivariate analysis controlling for Robson group and gestational age, with Holm correction for exploratory, indication-level, and Robson-group-specific comparisons.

## Methods

### Study design and patient cohort

We conducted a retrospective analysis of all cesarean sections at University Hospital Giessen and Marburg (UKGM), Giessen site, Germany, from January 1, 2015, to December 31, 2021. The patient cohort was compiled using operative schedules and birth registers. Ethics committee approval was obtained (reference number 75/21).

### Variables

Cesarean sections were classified as elective (pre-labour) or non-elective (decided during labour, including both planned intervention for a developing complication and emergency delivery for acute compromise), based on the documented decision timepoint. This classification agreed with the institution’s organizational primary/secondary/emergency label in 93.0% of records; the discrepant cases (7.0%) were retained under the decision-timepoint classification, as this corresponds directly to the clinical question of whether a decision was made before or during labour, independent of subsequent organizational documentation.

Among non-elective cesarean sections, threshold-susceptible ("soft") indications were defined a priori as protracted labour/arrest (overall, and separately for the first and second stage of labour) and non-reassuring cardiotocography, diagnoses considered susceptible to subjective threshold-setting in clinical practice. Protracted labor/arrest and its components (first-stage, second-stage protraction, arrest of progression) were derived from separate documented variables; in clinical practice these diagnoses are not always sharply distinguished, and the individual-level estimates for these components should be interpreted with this in mind. The composite soft-indicator result does not depend on this distinction. Guideline-defined absolute ("hard") indications, per the S3 guideline on cesarean section (AWMF 015–084), were defined as transverse lie, uterine rupture, placenta previa, and placental abruption, constellations in which vaginal delivery cannot be safely offered irrespective of physician judgment; together these account for <10% of all cesarean sections per the guideline. Cord prolapse, not named among the guideline’s four examples, but comparably acute and time-critical in obstetric practice, was analysed both as an extension of the hard indicator set and reported separately. Both sets were derived from separate, non-mutually exclusive variables and defined before any shift- or weekend-stratified analysis; 49 cesarean sections (1.6%) were documented with both a soft and a hard indication.

All cesarean deliveries were further classified according to the Robson Ten Group Classification System [11, 12], based on six obstetric parameters (parity, gestational age, number of fetuses, fetal presentation, onset of labour, and previous cesarean section), applied to account for differing baseline cesarean risk across obstetric populations and to enable international comparability; classification was complete for 99.9% of cases.

Temporal exposure was defined as weekday versus weekend (Monday–Friday vs. Saturday–Sunday) and shift (day shift, 08:00–16:00, 8 h, vs. evening/night shift, 16:00–08:00, 16 h). Shift assignment was based on the recorded time of delivery rather than the time of decision; cesarean sections decided shortly before a shift change, but delivered afterward may, therefore, be misclassified into the following shift, a non-differential misclassification expected to attenuate rather than inflate the estimated shift effect.

### Data sources and measurement

Maternal characteristics, fetal parameters, and documented cesarean section indications were extracted from hospital information systems. The documented basis for the decision was categorized as physician present at the bedside, physician informed remotely (e.g., by a midwife or colleague), a pre-planned antenatal discussion, or a diagnostic action without explicit presence information. Physician seniority at the time of decision (resident without senior consultation, specialist, attending, or chief physician) was derived from the variable Arzt, including cases with documented consultation with a more senior colleague.

Institutional practice during the study period was as follows. Cesarean delivery on maternal request was performed after a two-step informed consent process with a mandatory reflection period between the two counselling sessions. Trial of labour after cesarean was actively offered, and induction of labour was performed in Robson Group 5 following risk counselling, despite off-label status of several agents in this context. For breech presentation, external cephalic version, planned cesarean section, and vaginal breech delivery were all discussed and offered; vaginal breech delivery was reserved for favourable constellations, with pelvimetric MRI performed from 36 to 37 weeks of gestation to assess pelvic dimensions. The on-call shift model remained unchanged throughout the study period; a change of department leadership, with partial turnover of senior attending physicians, occurred in 2018.

### Study design and analytic approach

A rigorous test of whether the threshold for non-elective cesarean section varies with staffing-related temporal factors would require the underlying population at risk, i.e., all women in labour including those who delivered vaginally, as the analytic denominator. Such data were not available for this retrospective, cesarean-focused dataset: institutional labour ward statistics do not permit resolution by time of day, and Robson group is not recorded in either the electronic birth-registration system or the paper birth logbook, precluding reconstruction of a Robson-stratified at-risk denominator. We, therefore, restricted the primary analysis to non-elective cesarean sections and examined, conditional on a non-elective cesarean section having occurred, whether the character of the decision (indication type, cervical dilation at decision, and use of fetal scalp blood sampling as confirmatory diagnostics) differed by shift and day of week; this conditioning may introduce collider selection if the case mix of non-elective cesarean sections itself differs by time, which we address in the Limitations. Objectively acute indications (cord prolapse, placental abruption, uterine rupture), which cannot be deferred and should, therefore, show no temporal variation, served as a supportive comparison, though not a formal negative control for the analytic approach. Elective cesarean sections were analysed separately and reported descriptively as scheduling logistics rather than as evidence of temporal influence on decision-making.

### Statistical analysis

Poisson regression was used only for the descriptive characterization of elective cesarean section timing, accounting for unequal exposure time (1,825 weekdays vs. 730 weekend days; 20,456 day-shift hours vs. 40,912 evening/night-shift hours over the 7-year study period): glm(n ~ Exposure + offset(log(Exposure time)), family = poisson), where n is the count of elective cesarean sections per stratum and Exposure time is the corresponding total days (weekday/weekend comparison) or hours (shift comparison) of facility-time exposure, yielding rate ratios.

All primary and exploratory comparisons among non-elective cesarean sections were estimated using logistic regression models of the general form glm(Outcome == "Yes" ~ Exposure + Robson Group + GA, family = binomial), where Outcome denotes the indicator variable of interest (any soft indicator, any hard indicator, or an individual indication), Exposure denotes Shift (reference: day shift) or Weekend (reference: weekday), Robson Group is a 10-level factor, and GA is gestational age in weeks (continuous). This specification was used identically for the two pre-specified primary comparisons (effect of shift and of weekend on soft-indicator occurrence), reported without adjustment for multiple testing, and for the individual soft-indicator (*n* = 5 per family), hard-indicator (*n* = 6 per family), and exploratory full-indication-spectrum (*n* = 21 per family) comparisons, all Holm-corrected within their respective family, differing only in the outcome variable.

Two extensions to this base specification were used for specific analyses. The decision-context model replaced the temporal exposure with Decision context (reference: physician present at bedside): glm(Soft indicator == "Yes" ~ Decision context + Robson Group + GA, family = binomial). The staffing-interaction model included an interaction between shift and attending-physician involvement: glm(Soft indicator == "Yes" ~ Shift * Attending or above + Weekend + Robson Group + GA, family = binomial). The secular-trend model added calendar year as a linear term and a binary indicator for the documented change in department leadership: glm(Soft indicator == "Yes" ~ Shift + Weekend + Robson Group + GA + Year + Post-2018, family = binomial), where Post-2018 equals 1 for years from 2018 onward.

Associations between categorical variables were assessed using Fisher’s Exact Test, and the Wilcoxon test was used for metric variables; metric variables are summarized as median (IQR) and mean ± SD.

Analyses were performed in R version 4.5.2 [13]; figures were produced with ggplot2 [14] and descriptive summaries with the Hmisc package [15]. During data analysis and manuscript preparation, a large language model (Claude, Anthropic) was used for language editing, text structuring, and verification of R code; no patient data were provided to the tool, and all interpretations and conclusions remain the authors’ responsibility.

## Results

### Patient cohort

Of 5,850 cesarean sections performed between 2015 and 2021, decision timepoint was documented for 5,849 (99.98%); one case with missing data was excluded from classification. Cesarean sections were classified as elective (*n* = 2,851, 48.7%) or non-elective (*n *= 2,998, 51.2%) accordingly. Elective and non-elective cesarean sections differed across all recorded maternal and fetal characteristics except birth weight and length (all *p* < 0.001, Supplementary Table S1), consistent with their differing clinical context; elective cesarean sections occurred at somewhat earlier gestational age (median 38.0 vs. 38.4 weeks) with correspondingly higher Apgar scores (median 5-min Apgar 10 vs. 9). All analyses were adjusted for Robson group, complete for 99.9% of cases, to account for differing baseline case mix.

Within the non-elective cohort, maternal and fetal characteristics were largely similar by shift and by weekday (Table 1). Gestational age was slightly higher during evening/night shifts (median 38.4 vs. 38.1 weeks, *p* = 0.006) and on weekends (median 38.6 vs. 38.3 weeks, *p* = 0.006). Umbilical artery pH was marginally lower during evening/night shifts (median 7.280 vs. 7.294, *p* < 0.001, both median 7.30) and on weekends (median 7.30 vs. 7.29, *p* = 0.006). Given the small absolute magnitude of these differences relative to the physiological range, they are not considered clinically material on their own, but are reported for completeness.

**Table 1:** Maternal and Fetal Characteristics of non-elective cesarean sections), by shift (day vs. evening/night) and by weekday/weekend

Parameter	All	Day shift (*N* = 1,245)	Evening/night (*N* = 1,753)	*P*, shift	Weekday (*N* = 2,264)	Weekend (*N* = 734)	*P*, weekend
Maternal age, years	27/31/35 (30.9±5.6)	27/31/35 (31.0±5.6)	27/31/35 (30.7±5.5)	0.13	27/31/35 (30.9±5.6)	27/31/34 (30.8±5.5)	0.96
Gestational age, weeks	35.6/38.4/40.1 (37.3±3.9)	35.3/38.1/40.0 (37.1±3.9)	35.7/38.4/40.1 (37.4±3.9)	0.006	35.6/38.3/40.0 (37.2±3.8)	35.6/38.6/40.3 (37.3±4.0)	0.10
Birth weight, g	2370/3050/3550 (2898±890)	2340/3025/3530 (2887±910)	2380/3070/3556 (2905±876)	0.41	2365/3040/3550 (2896±888)	2395/3070/3555 (2902±899)	0.68
Birth length, cm	47/50/53 (49.3±5.1)	47/50/53 (49.2±5.1)	47/50/53 (49.4±5.1)	0.14	47/50/53 (49.3±5.1)	47.5/50/53 (49.6±5.0)	0.19
Head circumference, cm	32.5/34.0/35.0 (33.5±3.0)	32.5/34.0/35.5 (33.5±3.1)	32.5/34.0/35.0 (33.5±3.0)	0.72	32.5/34.0/35.0 (33.5±3.1)	32.5/34.0/35.0 (33.6±2.9)	0.92
Umbilical artery pH	7.25/7.30/7.33 (7.286±0.075)	7.26/7.30/7.34 (7.294±0.071)	7.25/7.29/7.33 (7.280±0.077)	<0.001	7.25/7.30/7.34 (7.287±0.077)	7.25/7.29/7.33 (7.280±0.071)	0.006
Umbilical vein pH	7.29/7.33/7.36 (7.319±0.072)	7.29/7.33/7.37 (7.324±0.068)	7.28/7.32/7.35 (7.315±0.073)	<0.001	7.29/7.33/7.36 (7.321±0.071)	7.28/7.32/7.36 (7.312±0.073)	0.012
Apgar score (5 min)	9/10/10 (9.3±1.1)	9/10/10 (9.3±1.2)	9/10/10 (9.3±1.1)	0.29	9/10/10 (9.3±1.1)	9/10/10 (9.4±1.0)	0.039

Data are presented as 1st quartile/median/3rd quartile (mean ± SD) for the non-elective cohort (*N* = 2,998), by shift (day vs. evening/night) and by weekday/weekend. Group differences were assessed using the Wilcoxon rank-sum test. Non-missing N per variable: maternal age and gestational age, *N* = 2,998; birth weight, *N* = 2,997; birth length, *N* = 2,848; head circumference, *N* = 2,854; umbilical artery pH, *N* = 2,914; umbilical vein pH, *N* = 2,440; Apgar score, *N* = 2,994. Missing values reflect incomplete documentation in the source hospital information systems and were not imputed

Before testing for temporal variation, we confirmed that the soft/hard classification behaved as expected across the elective/non-elective distinction: soft indicators were markedly more common among non-elective than elective cesarean sections (54.0% vs. 7.1%), as expected, whereas guideline-defined hard indicators occurred at similar rates in both groups (7.7% vs. 7.6%, *p* = 0.92), consistent with these indications arising independently of the elective/non-elective classification (Supplementary Table S1). We then assessed, among non-elective cesarean sections, whether the frequency of these indications varied by shift and by weekday/weekend.

### Decision character of non-elective cesarean section

Among non-elective cesarean sections, the pre-specified primary comparison found significantly higher adjusted odds of a threshold-susceptible indication (protracted labour/arrest or non-reassuring cardiotocography) during the evening/night shift than the day shift (OR 2.63, 95%CI 2.20–3.14, *p* <0.001), adjusted for Robson group and gestational age. A weekend effect was also observed, though smaller in magnitude (OR 1.37, 95%CI 1.12–1.67, *p* = 0.002) (Table 2). Elective cesarean sections were concentrated on weekdays (95.3%) and during day shifts (88.9%), consistent with scheduling during staffed working hours.

**Table 2:** Threshold-Susceptible (“Soft”) and Guideline-defined absolute (“Hard”) Indicators in Non-Elective CS

Indicator	Day (*N* = 1,245)	Night (*N* = 1,753)	OR shift (95% CI)	Weekday (*N* = 2,264)	Weekend (*N* = 734)	OR weekend (95% CI)	Holm-*p* (shift/week)
Any soft indicator (primary)	**42.8% (533)**	**62.0% (1,087)**	**2.63 (2.20–3.14)**	**52.2% (1,182)**	**59.7% (438)**	**1.37 (1.12–1.67)**	**<0.001/0.002**
Protracted labour/arrest	23.4% (291)	35.2% (617)	1.76 (1.46–2.13)	28.8% (652)	34.9% (256)	1.24 (1.01–1.53)	<0.001/0.125
Protracted first stage	16.6% (207)	23.0% (403)	1.40 (1.15–1.72)	20.1% (454)	21.3% (156)	0.97 (0.78–1.21)	0.001/0.902
Protracted second stage	6.7% (84)	12.2% (214)	1.73 (1.32–2.29)	8.7% (198)	13.6% (100)	1.53 (1.17–2.00)	<0.001/0.009
Labor arrest	20.7% (258)	31.1% (545)	1.66 (1.38–2.01)	25.0% (567)	32.2% (236)	1.35 (1.10–1.66)	<0.001/0.017
Non-reassuring CTG	28.4% (353)	39.4% (691)	1.68 (1.42–1.98)	34.3% (776)	36.5% (268)	1.07 (0.89–1.29)	<0.001/0.902
Any hard indicator (exploratory)	**8.4% (105)**	**7.2% (127)**	**0.99 (0.73–1.33)**	**8.3% (187)**	**6.1% (45)**	**0.77 (0.52–1.10)**	**0.923/0.155**
Hard indicator extended	8.6% (107)	7.7% (135)	1.04 (0.78–1.39)	8.7% (196)	6.3% (46)	0.74 (0.51–1.06)	0.906/0.423
Transverse lie	1.7% (21)	1.2% (21)	0.59 (0.23–1.52)	1.6% (37)	0.7% (5)	0.30 (0.08–1.02)	0.906/0.334
Uterine rupture	2.7% (34)	2.9% (50)	1.32 (0.84–2.10)	3.0% (68)	2.2% (16)	0.76 (0.42–1.30)	0.906/1.000
Placenta previa	2.3% (29)	0.9% (15)	0.39 (0.20–0.73)	1.8% (40)	0.5% (4)	0.31 (0.09–0.79)	0.023/0.177
Abruption	2.2% (27)	2.6% (46)	1.30 (0.80–2.15)	2.3% (53)	2.7% (20)	1.20 (0.69–2.03)	0.906/1.000
Cord prolapse	0.2% (2)	0.5% (8)	0.4% (9)	0.1% (1)	3.17 (0.78– 21.33)	0.38 (0.02–2.05)	0.748/1.000

Adjusted for Robson group and gestational age (logistic regression; model specification as described in Methods, Statistical Analysis). “Any soft indicator” is the pre-specified primary comparison, reported without multiplicity adjustment. All hard-indicator comparisons, including “any hard indicator”, are supportive, exploratory comparisons and Holm-corrected within their family (*n* = 6); remaining soft-indicator rows are exploratory and Holm-corrected within their family (*n* = 5). “Hard indicator” comprises the guideline-strict set (transverse lie, uterine rupture, placenta previa, abruption; AWMF 015–084); “hard indicator extended” adds cord prolapse, which is not named among the guideline’s four absolute-indication examples, but is comparably acute and time-critical in obstetric practice

**Fig. 1:**
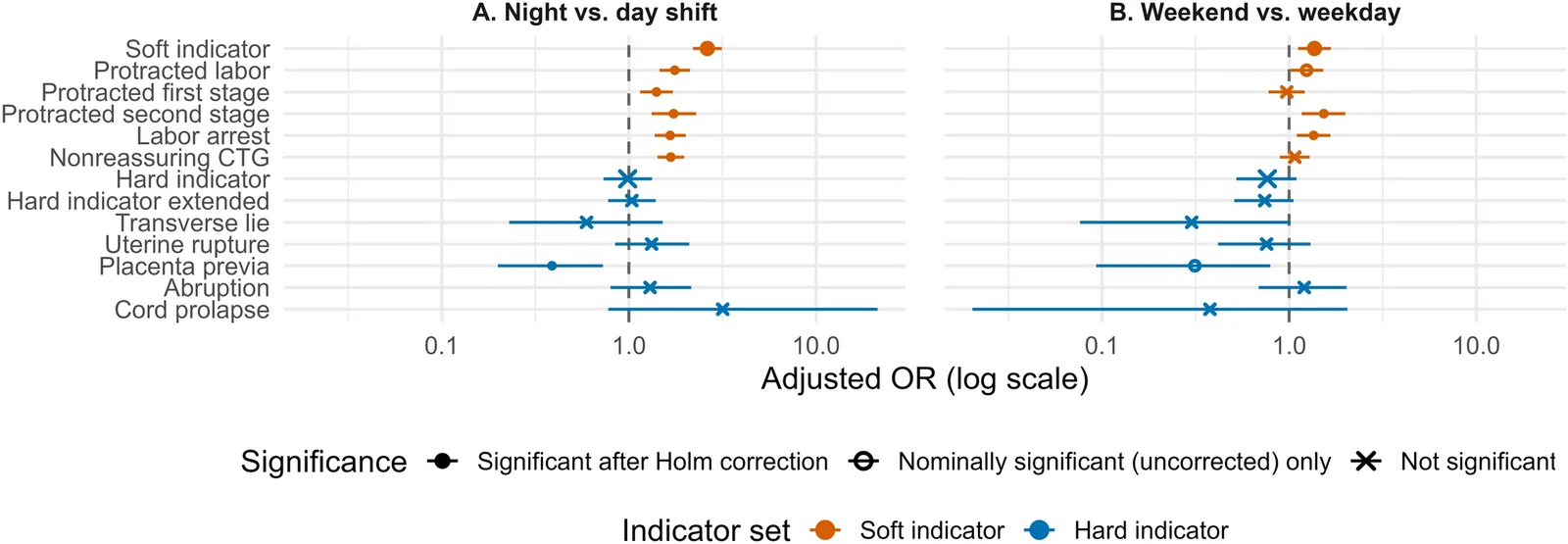
Adjusted odds ratios (95% CI, log scale) for threshold-susceptible ("soft", orange) and guideline-defined absolute ("hard", blue) indicators among non-elective cesarean sections, by (**A**) shift (evening/night vs. day) and (**B**) weekday/weekend, adjusted for Robson group and gestational age (logistic regression; model specification as described in Methods, Statistical Analysis). OR greater than 1 indicate a higher proportion of cesarean sections with the respective documented indicator during the evening/night shift (panel A) or on weekends (panel B), relative to the day shift or weekday reference category. "Hard indicator" comprises the guideline-strict set (transverse lie, uterine rupture, placenta previa, abruption; AWMF 015–084); "hard indicator extended" adds cord prolapse, which is not named among the guideline’s four absolute-indication examples, but is comparably acute and time-critical in obstetric practice. Bold point indicates the pre-specified primary comparison (any soft indicator), reported without multiplicity adjustment. Symbol shape indicates significance: filled circles remain significant after Holm correction within their respective family (*n* = 5 for soft, *n* = 6 for hard indicators); open circles are nominally significant (uncorrected *p* < 0.05) only; crosses are not significant. Dashed vertical line indicates no effect (OR=1)

Cord prolapse (10 events overall) is reported as a separate estimate. P values for the hard indications are Holm-corrected within a family of six comparisons (hard indicator extended, shown in Figure 1; transverse lie; uterine rupture; placenta previa; abruption; cord prolapse); P values for “any hard indicator” are unadjusted

The pattern for non-elective CS was consistent across all five individual threshold-susceptible indications for shift (protracted labour OR 1.76, protracted first stage OR 1.40, protracted second stage OR 1.73, labour arrest OR 1.66, non-reassuring CTG OR 1.68; all Holm-adjusted *p* ≤ 0.001) and for two of five for weekend (protracted second stage OR 1.53, Holm-*p* = 0.009; labour arrest OR 1.35, Holm-*p*=0.017; protracted labour OR 1.24, Holm-*p*=0.125, not significant after correction; protracted first stage and non-reassuring CTG showed no significant weekend association) Fig. 1.

The character of physician involvement documented at the time of decision was itself associated with the soft-indicator outcome (*p* < 0.001, Table 3). Relative to cases where the physician was present at the bedside, the adjusted odds of a threshold-susceptible indication were lower when the physician was informed remotely (OR 0.66, 95%CI 0.53–0.84, *p* = 0.001) and higher when the documented basis was a diagnostic action without explicit presence information (OR 1.45, 95%CI 1.17–1.81, *p* = 0.001). A small subgroup (n=88, 1.5% of the cohort) was documented as based on an antenatal planning discussion despite an intrapartum decision timepoint; these cases showed the lowest soft-indicator odds (5% vs. 54% among bedside-present cases), consistent with risk constellations discussed antenatally, but formally decided only once labour occurred.

**Table 3:** Decision Context and Provider Seniority

Term	Adjusted OR (95% CI)	*P*
Informed remotely (ref: present at bedside)	0.66 (0.53–0.84)	0.001
Diagnostic action only (ref: present at bedside)	1.45 (1.17–1.81)	0.001
Shift, without attending involvement	3.88 (2.24–6.78)	<0.001
Shift × attending-or-above interaction	0.64 (0.36–1.15)	0.14

An attending physician or above was involved in 90% of non-elective cesarean sections, with no significant difference by shift (91% day vs. 89% night, *p* = 0.12), but with fewer chief-physician-level decisions on weekends (2% vs. 5% on weekdays; overall distribution *p* < 0.001, Table 3). In an exploratory model, the shift effect on soft-indicator occurrence was estimated at OR 3.88 (95%CI 2.24–6.78) in cases without attending involvement; the interaction between shift and attending involvement was not significant (OR 0.64, 95%CI 0.36–1.15, *p* = 0.14), so this dataset does not establish whether attending-level seniority modifies the shift effect. A modest linear decline in soft-indicator odds was observed across the study period (adjusted OR 0.91 per year, 95%CI 0.83–0.99, *p* = 0.033); no significant discontinuity was detected at the documented change in department leadership in 2018 (adjusted OR 1.20, 95%CI 0.85–1.70, *p* = 0.30), so these data do not establish whether this specific personnel change contributed to the temporal pattern reported here.

Decision-context model: glm(Soft_indicator == "Yes" ~ Decision context + Robson Group + GA, family = binomial), reference category: physician present at bedside. Staffing-interaction model: glm(Soft_indicator == "Yes" ~ Shift * Attending_or_above + Weekend + Robson Group + GA, family = binomial); the reported "shift, without attending involvement" term and the shift × attending-or-above interaction term are both taken from this single model. Both models fitted on the non-elective cohort (*N* = 2,998). *GA* gestational age in weeks (metric)

### Hard indications: a supportive comparison

The guideline-defined hard indicator set (transverse lie, uterine rupture, placenta previa, placental abruption) showed no significant shift effect (OR 0.99, 95%CI 0.73–1.33, *p*=0.92) and no significant weekend effect (OR 0.77, 95%CI 0.52–1.10, *p* = 0.16) in this supportive comparison (Table 2). Indications that cannot, by clinical necessity, be deferred or expedited would be expected to show no temporal variation; the absence of such variation here is consistent with, though does not independently establish, the pattern observed for soft indications.

One exploratory exception was observed after Holm correction: placenta previa showed lower odds during evening/night shifts (OR 0.39, 95%CI 0.20–0.73, Holm-*p*=0.023); the weekend estimate was in the same direction, but not significant after correction (OR 0.31, Holm-*p*=0.18). As placenta previa-related cesarean sections are frequently scheduled semi-electively once bleeding risk is identified, this finding may reflect residual scheduling behaviour within the non-elective category rather than a threshold effect and is discussed as a limitation of this comparison’s specificity rather than a challenge to the primary finding.

## Discussion

### Main findings

The rate of non-elective cesarean sections per unit facility-time was lower during evening/night shifts and, to a smaller extent, on weekends, a pattern that alone is compatible with either a staffing-related threshold effect or simply fewer births occurring during these periods. Because the appropriate population denominator was unavailable, we separately examined, among non-elective cesarean sections that occurred, whether the proportion with documented soft indications differed by shift and weekend. This proportion was higher during evening/night shifts and on weekends, even after adjustment for Robson group, gestational age, and multiple testing. This finding describes the documented indications among the cesarean sections that occurred and does not resolve the ambiguity in the overall rate.

### Comparison with literature

Our findings are consistent with prior work suggesting that the timing of unplanned cesarean section is influenced by factors unrelated to the underlying obstetric course. Costa-Ramón et al. found that physician demand for leisure time is a significant predictor of unplanned cesarean sections, differentiating indications with a clinical basis from a "gray area" of diagnoses comparable to our protracted labour/arrest and non-reassuring cardiotocography indications [5]. Using a population-based at-risk denominator unavailable to us, Son et al. more recently found that the frequency of intrapartum cesarean delivery rises throughout the day and declines at night, consistent with the lower descriptive, facility-time-adjusted rate we observed [6]. Our finding that the share of threshold-susceptible indications among cesarean sections that did occur was nonetheless higher at night suggests these findings are complementary: the overall frequency of intervention appears lower during evening/night shifts and weekends, but interventions that do occur skew toward discretionary indications, a pattern only detectable at the level of decision composition rather than frequency alone. Separately, the presence of senior obstetricians on the labour ward has been associated with only small differences in intervention rates and no significant differences in neonatal outcomes [7], our own data likewise did not establish whether attending-physician involvement modifies the shift effect. Our Robson-stratified, indication-level approach extends this literature by directly comparing threshold-susceptible against guideline-defined absolute indications within the same cohort, providing a built-in supportive comparison absent from prior studies based on time-of-day variation alone.

### Strengths and limitations

This study has several strengths. The large, seven-year, single-centre cohort (*N* = 5,850) with near-complete Robson classification (99.9%) and indication-level detail allowed adjustment for baseline case mix throughout. Non-elective cesarean sections were identified by documented decision timepoint rather than organizational labelling, which agreed with the latter in 93.0% of records; the discrepant cases were predominantly complications identified before labour onset, but organizationally filed as non-elective, and using the decision-timepoint variable avoids misclassifying these cases against the clinical question of interest. The pre-specified distinction between threshold-susceptible ("soft") and guideline-defined absolute ("hard") indications, informed by the S3 guideline on cesarean section (AWMF 015–084), provided a built-in supportive comparison: the limited temporal variation in hard indicators, alongside a consistent effect across all five soft indicators, is compatible with a difference confined to discretion-susceptible indications, rather than relying on a single, potentially fragile estimate. Multiple testing was addressed transparently, with two pre-specified primary comparisons reported unadjusted and all group-specific and exploratory comparisons Holm-corrected within their respective family.

Several limitations should be considered. First, a population-based at-risk denominator (all women in labour, including those delivered vaginally) was not available in this dataset. We addressed this by comparing the character rather than the frequency of non-elective cesarean sections, an approach that does not require the total-birth denominator, but cannot fully distinguish a genuinely lower intervention threshold from a shift in the composition of cases proceeding to cesarean section; this conditioning may introduce collider selection if the underlying case mix of non-elective cesarean sections itself differs by time. Second, the guideline-strict hard indicator set showed one exception, placenta previa, with lower odds during evening/night shifts after correction; this likely reflects semi-elective management once bleeding risk is identified rather than a true threshold effect, but it qualifies the specificity of this comparison. Third, cardiotocography findings were documented as a binary category without a distinct "suspicious" subcategory in routine clinical documentation, precluding the finer distinction between suspicious and pathological tracings that would be clinically relevant to a threshold hypothesis. Fourth, fetal scalp blood sampling was used inconsistently across attending physicians during the study period, limiting statistical power for this measure. Fifth, this is a single-centre study; while a documented change in department leadership occurred in 2018, we found no evidence of an associated discontinuity in soft-indicator occurrence, and a modest linear decline across the study period was observed instead. Unmeasured institutional changes over the seven-year period cannot, however, be excluded. Finally, an exploratory analysis of a further 21 indications not included in the pre-specified soft/hard sets (Supplementary Table S2, Supplementary Figure S1) identified one additional, guideline-classified discretionary diagnosis (malposition) with a convergent pattern; as this was not pre-specified, it is reported as hypothesis-generating and requires independent confirmation.

### Clinical implications

These findings suggest the composition of documented indications for non-elective cesarean section differs across shifts and, to a lesser extent, across weekdays and weekends, with discretion-susceptible indications documented more often during evening/night shifts and weekends than indications with limited clinical discretion, providing a specific and testable signal for quality improvement rather than a generic staffing complaint. The shift effect on soft-indicator occurrence did not differ significantly by attending-physician involvement (interaction p=0.14); this dataset, therefore, does not establish whether attending involvement modifies the pattern. The character of physician presence at the time of decision, being at the bedside versus informed remotely, was independently associated with indication type, a documentation pattern that institutional audit could further characterize. From a resource perspective, cesarean delivery requires an operating team, anaesthesia, and postpartum/neonatal care capacity that is comparatively scarcer outside day-shift hours; if the pattern observed here reflects a resource availability rather than a genuine change in clinical judgment, it would support institutional review of decision-making protocols and second-opinion procedures for discretionary indications during evening/night shifts and weekends, rather than an assumption that indication composition is uniform across the day. Given the unresolved denominator limitation, these implications should be regarded as motivating institutional audit and, ideally, prospective or population-denominator studies, rather than as directly establishing a causal staffing effect on clinical decision-making.

## Supplementary Information

Below is the link to the electronic supplementary material.

Supplementary file1 (TIFF 667 KB)

Supplementary file2 (DOCX 16 KB)

Supplementary file3 (DOCX 19 KB)

## Data Availability

The datasets analyzed are available from the corresponding author upon reasonable request, subject to institutional privacy regulations.
