# Supplementary material for: Temporal influences in non-elective cesarean sections and indication patterns
Source: Arch Gynecol Obstet. 2026 Nov 12;313(1):310. doi: 10.1007/s00404-026-08604-y (PMC13759544; doi:10.1007/s00404-026-08604-y)
Supplement: Supplementary file 1 [file 404_2026_8604_MOESM2_ESM.docx]

**Supplementary Table S1. Maternal, Fetal, and Indication Characteristics by Elective vs. Non-Elective Cesarean Section**

**A. Maternal and fetal characteristics**

| **Parameter** | **Elective (N=2,851)** | **Non-elective (N=2,998)** | **Combined (N=5,849)** | **P** |
| --- | --- | --- | --- | --- |
| Maternal age, years | 28/32/36 (31.9±5.5) | 27/31/35 (30.9±5.6) | 28/31/35 (31.4±5.6) | <0.001 |
| Gestational age, weeks | 37.0/38.0/38.6 (37.2±2.7) | 35.6/38.4/40.1 (37.3±3.9) | 36.3/38.1/39.1 (37.2±3.4) | <0.001 |
| Birth weight, g | 2585/3054/3452 (2951±786) | 2370/3050/3550 (2898±890) | 2480/3050/3505 (2923±841) | 0.37 |
| Birth length, cm | 48.0/50.0/52.0 (49.7±4.2) | 47.0/50.0/53.0 (49.3±5.1) | 48.0/50.0/52.0 (49.5±4.7) | 0.72 |
| Head circumference, cm | 33.0/34.8/36.0 (34.3±2.5) | 32.5/34.0/35.0 (33.5±3.0) | 33.0/34.5/35.5 (33.9±2.8) | <0.001 |
| Umbilical artery pH | 7.290/7.330/7.350 (7.316±0.068) | 7.250/7.300/7.330 (7.286±0.075) | 7.270/7.310/7.340 (7.300±0.074) | <0.001 |
| Umbilical vein pH | 7.330/7.360/7.380 (7.350±0.103) | 7.290/7.330/7.360 (7.319±0.072) | 7.310/7.340/7.370 (7.334±0.090) | <0.001 |
| Apgar score (5 min) | 9.0/10.0/10.0 (9.5±1.1) | 9.0/10.0/10.0 (9.3±1.1) | 9.0/10.0/10.0 (9.4±1.1) | <0.001 |

**B. Soft and hard indicator occurrence**

| **Indicator** | **Elective (N=2,851)** | **Non-elective (N=2,998)** | **Combined (N=5,849)** | **P** |
| --- | --- | --- | --- | --- |
| **Any soft indicator** | **7.1% (201)** | **54.0% (1,620)** | **31.1% (1,821)** | **<0.001** |
| **Any hard indicator** | **7.6% (218)** | **7.7% (232)** | **7.7% (450)** | **0.922** |

*Panel A: data are presented as 1st quartile/median/3rd quartile (mean ± SD) for the full cohort (N=5,849), by elective (pre-labour) vs. non-elective (intrapartum) classification; group differences assessed using the Wilcoxon rank-sum test. Panel B: percentages and counts denote the proportion of cesarean sections with at least one soft or hard indicator, respectively, within each classification; group differences assessed using Fisher's Exact Test.*
