# Supplementary material for: Temporal influences in non-elective cesarean sections and indication patterns
Source: Arch Gynecol Obstet. 2026 Nov 12;313(1):310. doi: 10.1007/s00404-026-08604-y (PMC13759544; doi:10.1007/s00404-026-08604-y)
Supplement: Supplementary file 2 [file 404_2026_8604_MOESM3_ESM.docx]

**Supplementary Table S2 Exploratory full indication spectrum (n=21 per family)**

|  | **OR night (95% CI)** | **p (raw)** | **Holm-p** | **OR weekend (95% CI)** | **p (raw)** | **Holm-p** |
| --- | --- | --- | --- | --- | --- | --- |
| Prior cesarean section | 0.65 (0.51–0.83) | <0.001 | 0.010 | 0.72 (0.53–0.96) | 0.025 | 0.509 |
| Breech presentation | 1.01 (0.67–1.53) | 0.973 | 1.000 | 1.07 (0.67–1.70) | 0.782 | 1.000 |
| Preeclampsia | 0.98 (0.68–1.44) | 0.930 | 1.000 | 0.97 (0.62–1.49) | 0.901 | 1.000 |
| HELLP syndrome | 0.72 (0.41–1.27) | 0.262 | 1.000 | 1.10 (0.56–2.05) | 0.763 | 1.000 |
| Suspected amniotic infection syndrome | 1.02 (0.74–1.41) | 0.916 | 1.000 | 0.95 (0.65–1.37) | 0.779 | 1.000 |
| Large for gestational age | 0.61 (0.46–0.81) | <0.001 | 0.012 | 0.71 (0.50–1.00) | 0.055 | 0.975 |
| Small for gestational age | 0.91 (0.56–1.47) | 0.692 | 1.000 | 1.24 (0.72–2.07) | 0.418 | 1.000 |
| Fetal growth restriction | 0.84 (0.58–1.22) | 0.367 | 1.000 | 0.96 (0.62–1.47) | 0.870 | 1.000 |
| Fetal malformation | 0.62 (0.44–0.87) | 0.005 | 0.091 | 0.62 (0.39–0.95) | 0.035 | 0.669 |
| Multiple pregnancy | 0.65 (0.09–2.95) | 0.610 | 1.000 | 0.51 (0.12–2.56) | 0.370 | 1.000 |
| Pathological Doppler finding | 0.65 (0.40–1.07) | 0.090 | 1.000 | 0.65 (0.33–1.19) | 0.187 | 1.000 |
| Malposition (lie/ presentation/position anomaly) | 1.44 (1.11–1.89) | 0.007 | 0.114 | 1.65 (1.26–2.15) | <0.001 | 0.005 |
| Cephalopelvic disproportion | 1.41 (0.99–2.02) | 0.061 | 0.919 | 1.04 (0.71–1.50) | 0.839 | 1.000 |
| Maternal exhaustion | 1.02 (0.74–1.41) | 0.895 | 1.000 | 0.85 (0.58–1.22) | 0.386 | 1.000 |
| Maternal request | 0.55 (0.34–0.90) | 0.018 | 0.289 | 1.17 (0.67–1.98) | 0.563 | 1.000 |
| Failed induction | 0.26 (0.19–0.33) | <0.001 | <0.001 | 0.74 (0.55–1.00) | 0.054 | 0.975 |
| Premature rupture of membranes | 0.94 (0.78–1.14) | 0.545 | 1.000 | 0.99 (0.80–1.23) | 0.959 | 1.000 |
| Preterm labour | 1.14 (0.85–1.54) | 0.384 | 1.000 | 0.78 (0.54–1.11) | 0.178 | 1.000 |
| Twin weight discrepancy | 1.73 (0.65–5.47) | 0.304 | 1.000 | 1.04 (0.33–2.80) | 0.941 | 1.000 |
| Pathological fetal scalp blood sample | 1.16 (0.58–2.38) | 0.683 | 1.000 | 0.87 (0.37–1.85) | 0.731 | 1.000 |
| Vaginal bleeding | 1.11 (0.75–1.66) | 0.596 | 1.000 | 0.76 (0.46–1.21) | 0.266 | 1.000 |

*Highlighted rows: Holm-significant in at least one exposure (Prior cesarean section, Large for gestational age, Malposition, Failed induction — shift; Malposition — weekend). Failed induction and prior cesarean section are discussed in the main text as likely artifacts of procedural timing (induction management and scheduled review of prior-cesarean cases are concentrated during day shifts) rather than decision-threshold effects. Malposition is discussed as a hypothesis-generating observation convergent with the primary finding.*
